# Amphipathic helical peptide-based fluorogenic probes for a marker-free analysis of exosomes based on membrane-curvature sensing†

**DOI:** 10.1039/d0ra07763a

**Published:** 2020-10-19

**Authors:** Yusuke Sato, Kazuki Kuwahara, Kenta Mogami, Kenta Takahashi, Seiichi Nishizawa

**Affiliations:** Department of Chemistry, Graduate School of Science, Tohoku University Sendai 980-8578 Japan yusuke.sato.a7@tohoku.ac.jp; JST, PRESTO 4-1-8 Honcho, Kawaguchi Saitama 332-0012 Japan

## Abstract

With increasing knowledge about the diverse roles of exosomes in the biological process, much attention has been paid to develop analytical methods for detection and quantification of exosomes. Immunoassays based on the recognition of exosomal protein markers by antibodies were widely used. However, considering that exosomal protein composition varies with the cell type, the protein markers should be carefully selected for a sensitive and selective analysis of target exosomes. Herein, we developed a new class of exosome-binding fluorogenic probes based on membrane curvature (MC) sensing of amphipathic helical (AH) peptides for exosome analysis without the need to use protein markers on the exosomal membranes. The C-terminal region of apolipoprotein A-I labeled with Nile red (ApoC-NR) exhibited a significant fluorescence enhancement upon selective binding to the highly curved membranes of synthetic vesicles. Circular dichroism (CD) measurements involving 1,2-dioleoyl-*sn-glycero*-3-phosphocholine (DOPC)/1-2-dioleoyl-*sn*-glycerol (DOG) vesicles suggested that ApoC-NR recognizes the lipid packing defects in the surface of highly curved membranes *via* the hydrophobic insertion of the α-helix structure of the ApoC unit. ApoC-NR exhibited a stronger binding affinity for exosome-sized vesicles and a higher MC selectivity compared to all other previously reported peptide probes. ApoC-NR can be used in a simple and rapid “mix and read” analysis of various kinds of exosomes derived from different cell types (limit of detection: –10^5^ particles/μL) without being influenced by the variation in the expression of the surface proteins of the exosomes, which stands in sharp contrast to immunoassays.

## Abstract 2

Fluorogenic probes based on membrane curvature sensing-amphipathic helical peptides have been developed for a marker-free exosome analysis.

## Graphical abstract

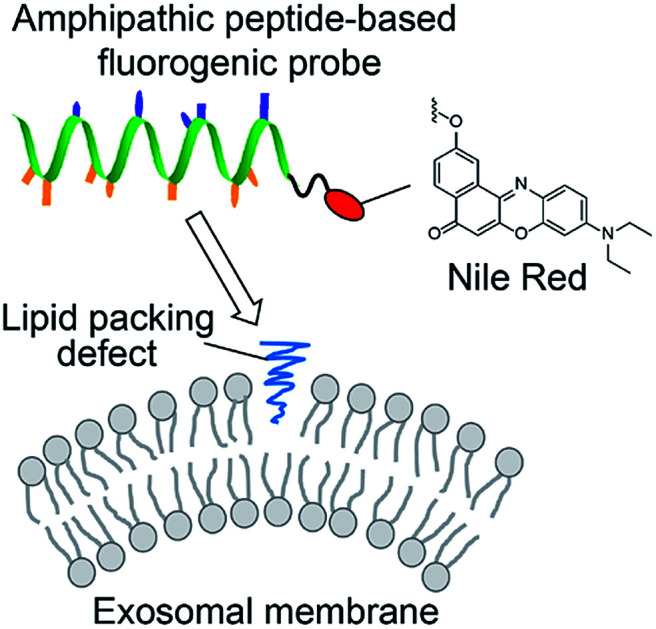

## Introduction

Exosomes, a subgroup of extracellular vesicles that generally range from 30 to 150 nm in diameter, have emerged as novel intercellular communication tools.^1^ They contain diverse proteins and genetic information reflective of their parent cells. There is increasing evidence that exosomes and their cargos have great potential in diagnostic and therapeutic applications.^2,3^ The determination of exosome concentrations is essential to realize these applications. Nanoparticle tracking analysis (NTA), where the light scattering of individual particles is analyzed, has been widely used to directly estimate exosome concentrations. The major drawbacks of NTA are the expensive apparatus and careful analysis required to ensure reliable results.^4^ Alternatively, the enzyme-linked immunosorbent assay (ELISA), which relies on the antibody recognition of surface protein markers such as CD63 and CD9, is more practical, as it only requires general lab equipment.^5,6^ However, its detection sensitivity depends in principle on the amount of protein markers. Considering that exosomal protein composition varies with the cell type, including the origin and the condition of the cells,^7–9^ a judicious selection of the protein markers to achieve a sensitive and selective analysis of target exosomes is required. Currently, there are no proteins that are known to be constitutively sorted into vesicles independent of the cell type, and the lack of invariant housekeeping markers hampers the comparative analysis of exosomes derived from different cell types.^10,11^ In addition, conventional ELISA suffers from time-consuming (>3 h) and laborious procedures that include washing and incubation steps. Moreover, the use of antibodies has intrinsic limitations given their low stability, high production cost, high batch-to-batch variation, and the difficulties associated with their modification. Although nucleic acid aptamers have emerged as useful replacements that can overcome these limitations,^8,9,12^ these assays still suffer from the aforementioned marker concerns.

**Fig. 1:**
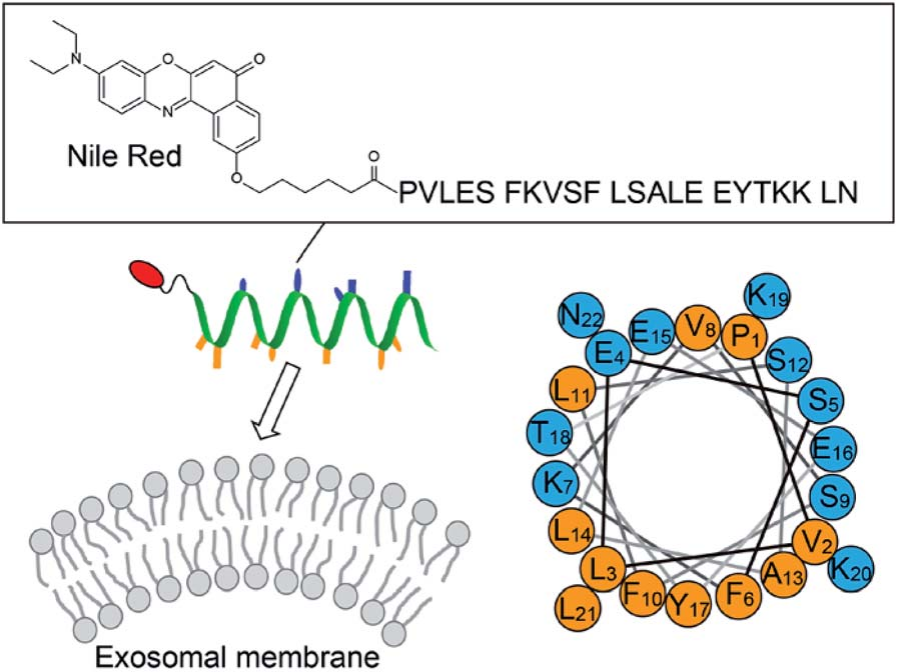
Schematic illustration of the exosome analysis with the AH peptide-based fluorescent probe ApoC-NR (*cf.* Fig. S1†). A helical wheel representation of ApoC sequence is also shown, wherein the hydrophobic and the polar residues are orange and blue, respectively.

This article describes the development of fluorescent molecular probes targeting lipid membranes for exosome analysis without the need to use protein markers (Fig. 1). Exosomal membranes are characterized by highly-curved surface due to the inherently small size compared to other extracellular vesicles such as microvesicles.^13^ This distinctive feature is considered to be a common trait in exosomes irrespective of the types of the parent cells. Targeting highly-curved membranes of exosomes by molecular probes thus provides a straightforward strategy for exosome analysis unbiased by different surface protein expression. Here, amphipathic helical (AH) peptides that can sense the membrane curvature (MC)^14,15^ were utilized for recognition of exosomal membranes (Fig. 1). AH peptides are increasingly studied for membrane remodeling^14,15^ and the manipulation of cell membranes,^16^ but they have as yet to be examined for the exosome analysis. AH peptides should be preferable to antibodies in immunoassays for exosome recognition, due to stability, availability and modifiability. AH peptides were coupled with environment-sensitive fluorophores in order to obtain the fluorogenic response upon binding to exosomes, which allows the “mix and read” analysis of target exosomes in homogeneous and wash-free format. The binding and fluorogenic functions of AH peptide probes are described as useful tools for the simple and rapid detection of various kinds of exosomes.

## Results and discussion

### Membrane curvature-dependent fluorogenic response

**Fig. 2:**
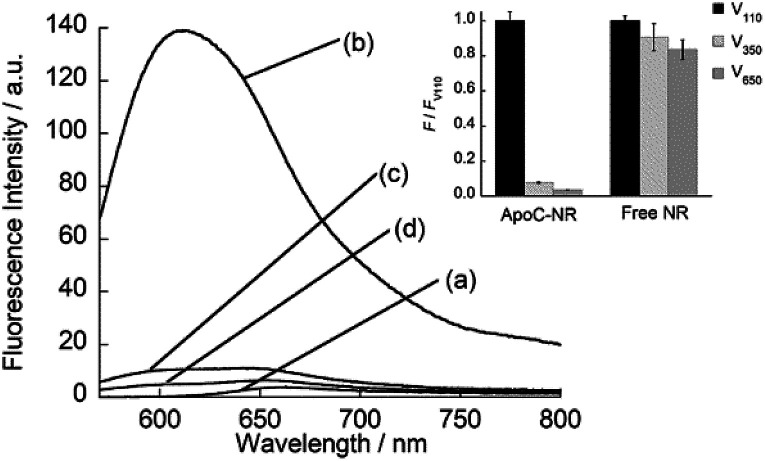
Fluorescence spectra of ApoC-NR in the (a) absence and presence of (b) V_110_, (c) V_350_, and (d) V_650_. Inset: Comparison of the fluorescence response for the vesicles between ApoC-NR and a free NR molecule. *F* and *F*_V_110__ denote the fluorescence intensity of the probe in the presence of synthetic vesicles and V_110_, respectively. Excitation, 552 nm. Analysis, 613 nm (ApoC-NR); 633.5 nm (free NR).

We designed the fluorogenic molecular probes composed of the C-terminal amphipathic region of human apolipoprotein A-I, corresponding to residues 220–241 in the full length protein (abbreviated as ApoC; Fig. 1) because of its preferential binding to curved membranes rather than flat membranes.^17,18^ Nile red (NR) was attached to the N-terminal of ApoC unit to afford ApoC-NR (Fig. 1 and S1†). ApoC-NR was synthesized by solid phase synthesis based on Fmoc chemistry (see ESI†). The crude product was purified by a reverse-phase HPLC system and the identity was verified by MALDI-TOF-MS (Fig. S2 and Table S1†). The fluorescence response of ApoC-NR was examined for synthetic vesicles of various sizes, whose lipid compositions are close to those of the plasma membranes of mammalian cells (50% POPC, 15% cholesterol, 15% POPE, 20% POPS)^19^ in PBS buffer (pH = 7.4) at 25 °C. Vesicles with diameters of 110 nm (V_110_) and 650 nm (V_650_) were used as representative models of the exosomes and the microvesicles, respectively. Vesicles with an intermediate diameter of 350 nm (V_350_) were also tested. As shown in Fig. 2, the NR unit of the probe (2.0 μM) showed a weak emission with a maximum at 663 nm. The addition of V_110_ ([total lipid] = 500 μM) caused a significant fluorescence enhancement as well as a blue shift of the emission peak (∼50 nm). This result suggests that the NR unit is in a hydrophobic environment, presumably partitioning into the membrane.^20,21^ This response is much larger than those for V_350_ and V_650_, suggesting the binding of ApoC-NR in a curvature-dependent manner. This is due to the contribution of the ApoC unit, given that an NR molecule without any peptides (Fig. S3†) lacked MC sensing ability (inset, Fig. 2).

### Binding affinity and mechanism

**Fig. 3:**
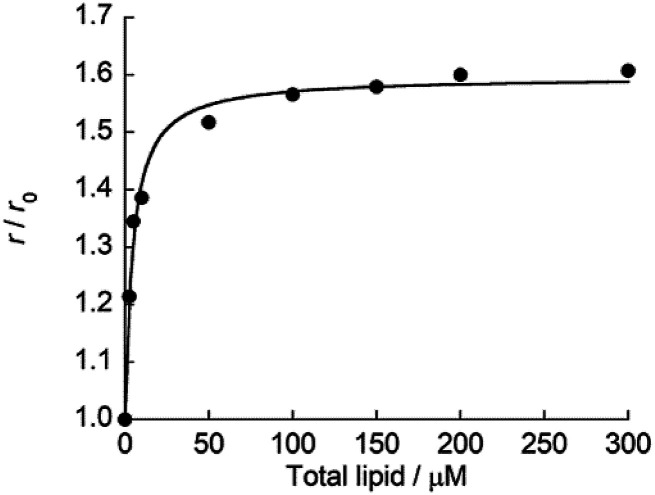
Titration curve for the binding of ApoC-NR to V_110_. [ApoC-NR] = 2.0 μM. *r* and *r*_0_ denote the fluorescence anisotropy of ApoC-NR in the presence of synthetic vesicles and absence of the vesicles, respectively. The obtained curve was analyzed with the fitting equation.^22^

**Fig. 4:**
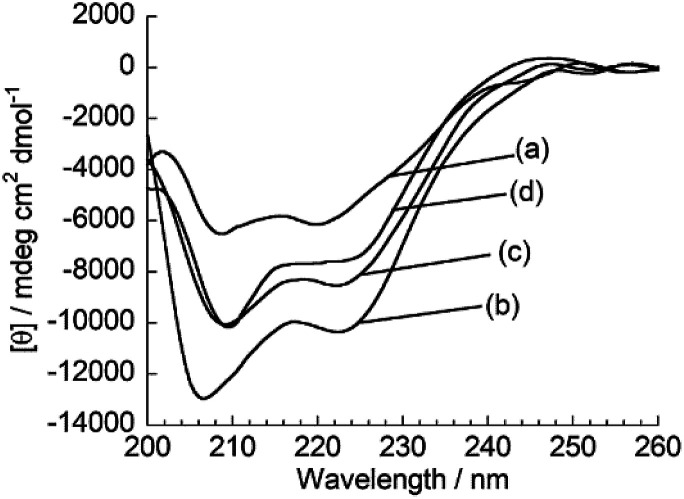
CD spectra of ApoC-NR in the (a) absence and presence of (b) V_110_, (c) V_350_ and (d) V_650_. [ApoC-NR] = 2.0 μM, [total lipid] = 100 μM.

Subsequently, fluorescence anisotropy titration experiments were performed in order to assess the binding affinity of ApoC-NR. The apparent dissociation constant (*K*_d_), which describes the lipid concentration where 50% of the probe is bound,^22^ was determined as 4.7 ± 0.61 μM for V_110_ (Fig. 3). This affinity is 17-fold and 22-fold stronger than those for V_350_ and V_650_, respectively (Fig. S4:†*K*_d_/μM; V_350_, 82 ± 12; V_650_, 100 ± 8.2). Apparently, ApoC-NR exhibits strong and selective binding to highly curved membranes. Circular dichroism (CD) measurements were done in order to explore the molecular basis of the binding of ApoC-NR (Fig. 4). α-Helix content of ApoC-NR was estimated as 17% according to the literature using the signal intensity at 222 nm.^23^ We found that the binding of ApoC-NR to V_110_ resulted in the increase in the α-helix content (30%), indicating that the ApoC unit is induced to fold into α-helix structure upon binding to V_110_. The α-helix content of ApoC-NR for V_110_ is higher than those for V_350_ (25%) and V_650_ (22%), reflecting the higher affinity of ApoC-NR toward highly-curved membranes. These results suggest that the binding of ApoC-NR is primarily driven by insertion of hydrophobic face in α-helical ApoC unit into the lipid packing defects that result from the mismatch between the actual MC and the lipid geometry (hydrophobic insertion).^14,15^ This notion was further supported by examining the binding to 1,2-dioleoyl-*sn-glycero*-3-phosphocholine (DOPC)/1-2-dioleoyl-*sn*-glycerol (DOG) vesicles whose size is similar to that of the previously tested vesicles (Fig. S5†). It has been previously reported that the introduction of cone-shaped DOG into a DOPC vesicle induces lipid packing defects.^24^ Accordingly, we prepared vesicles with varying DOPC/DOG ratios, and observed that the α-helicity in the bound state and the fluorescent response of ApoC-NR increased with increasing DOG content. Similar to other AH peptides,^25,26^ this can be interpreted by an acceleration of ApoC-NR binding to the vesicles due to increased lipid packing defects. The recognition of the lipid packing defects is responsible for the binding selectivity of ApoC-NR toward highly curved membranes (*cf.*Fig. 2). Note that the selectivity is markedly reduced in a mutant probe containing a helix-breaking proline residue (ApoCmut-NR; Fig. S1 and S6†).^27^ This underlines the importance of the propensity of the peptide sequence to form an α-helix.

### Comparison with other MC sensing peptide-based probes

**Fig. 5:**
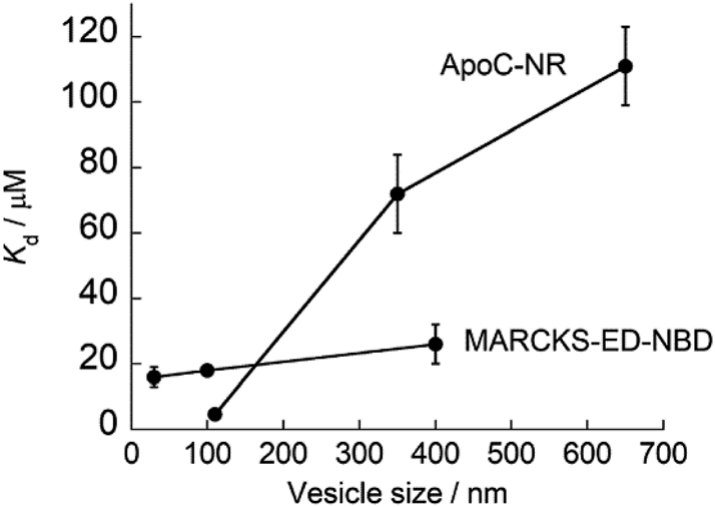
Dependence of the *K*_d_ values of ApoC-NR and MARCKS-ED-NBD^23^ on the size of the synthetic vesicles.

As described above, ApoC-NR functions as a useful fluorogenic probe with MC sensing ability, which stands in contrast to typical exosomal membrane-binding fluorescent probes, such as carbocyanine dyes, which simply insert into the membrane regardless of its curvature.^28,29^ In this context, Yin group has developed several MC sensing peptide-based fluorescent probes for exosome analysis.^30–32^ Of these, the MARCKS-ED (the effector domain of a myristoylated alanine-rich C-kinase substrate) labeled at the N-terminal with an NBD dye (MARCKS-ED-NBD: NBD-KKKKK RFSFK KSFKL SGFSF KKNKK) exhibited the strongest binding affinity through electrostatic interactions.^30^ Significantly, we found that the affinity of ApoC-NR for the exosome-sized vesicles (V_110_) was one order of magnitude higher than MARCKS-ED-NBD (*K*_d_ = 18 μM)^30^ for similarly sized vesicles. Moreover, the MC selectivity of ApoC-NR was remarkably superior to that of MARCKS-ED-NBD, as clearly demonstrated by the dependence of *K*_d_ values on the vesicle size (Fig. 5). It was also higher than that of any other previously reported MC-sensing peptide probe (Table S2†). This is not due to the difference in the fluorophore, as high MC selectivity was also observed in the NBD-carrying probe, ApoC-NBD (Fig. S7†). Instead, this selectivity must arise from the distinct binding modes of the probes. The electrostatic interactions that primarily drive the binding of the MARCK-ED-NBD do not depend on the MC.^33,34^ Conversely, the binding of ApoC-NR is based on the recognition of lipid packing defects that were encountered more frequently in highly curved membranes than the membranes with low curvature (*cf.*Fig. 2). It is thus highly likely that AH peptides are useful scaffolds for the selective targeting of highly curved exosomal membranes. In contrast to the case of MARCKS-ED-NBD, the removal of the anionic phospholipid POPS from the vesicles did neither impair the strong affinity nor the MC selectivity of ApoC-NR (Table S2†). ApoC-NR can thus be applied to the analysis of exosomes that contain low levels of POPS such as those derived from reticulocytes and adipocytes^35,36^ as well as those collected from seminal fluid.^37^

### Application to exosome analysis

**Fig. 6:**
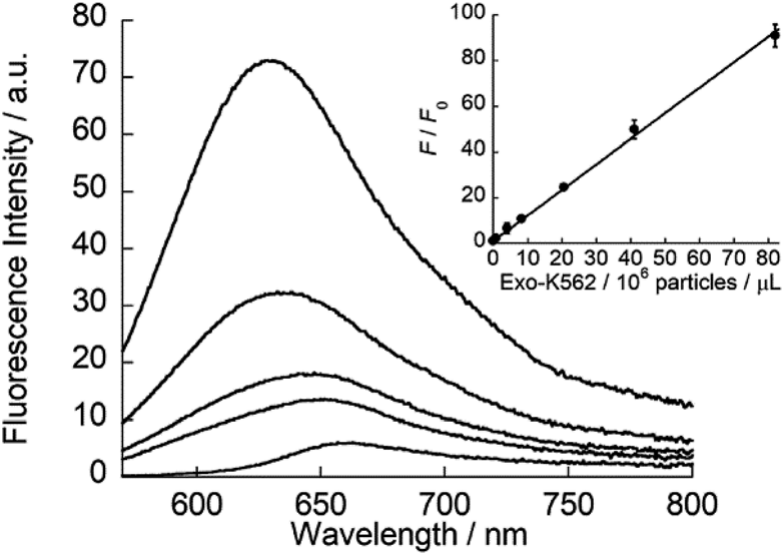
Fluorescence response of ApoC-NR (2.0 μM) for Exo-K562 (0.082–8.2 × 10^7^ particles/μL). Inset: calibration curve of Exo-K562 based on the fluorescence response (*F*/*F*_0_) of ApoC-NR. *F* and *F*_0_ denote the fluorescence intensity of ApoC-NR at 620 nm in the presence and absence of Exo-K562, respectively. Excitation, 552 nm.

ApoC-NR was finally applied to the fluorescence detection of exosomes derived from K562 cells (Exo-K562; Fig. S8†). As shown in Fig. 6, ApoC-NR exhibited a fluorescence enhancement for Exo-K562 in a “mix and read” procedure. As observed for synthetic vesicles (*cf.*Fig. 3), the α-helix content of ApoC-NR increased upon binding to Exo-K562 (Fig. S9†). A good linear relationship was observed between the fluorescence response of ApoC-NR and the exosome concentration. The limit of detection (LOD) was estimated to be 5.3 × 10^5^ particles/μL. Although this value is comparable to that of ELISA^38,39^ (LOD = 10^5^–10^7^ particles/μL), the present assay exhibits the distinct advantages of simplicity and rapid analysis (within 5 min). In addition, we observed a concentration-dependent fluorescence response of ApoC-NR for exosomes from BPH-1 (Exo-BPH-1), U87MG (Exo-U87MG) and A549 (Exo-A549) cells (Fig. S10†). Notably, the LOD values for these exosomes were comparable to that for Exo-K562 (LOD/10^5^ particles/μL: Exo-BPH-1, 2.1; Exo-U87MG, 3.2; Exo-A549, 1.8) although surface protein profiles of these exosomes largely differ from each other.^40^ These results clearly show that ApoC-NR can serve as a versatile tool for the exosome quantification without being influenced by the variation in the expression of surface proteins. Marker-free exosome quantification has previously been achieved based on colorimetric detection of acetylcholinesterase activity. However, this method requires a longer assay time (30 min)^29^ compared to our assay.

## Conclusions

In summary, a new class of exosomal membrane-binding fluorogenic probes was developed based on the MC-sensing ability of AH peptides for a marker-free analysis of exosomes. ApoC-NR can function as versatile tool for the exosome quantification as its detection sensitivity is hardly influenced by the variation in the expression of surface proteins. The simple and rapid quantification of exosomes in our system provide benefits in diagnostic applications. In order to make the assay more practical, further efforts are needed to enhance the binding affinity of the probes for the improved detection sensitivity. For instance, the intramolecular crosslinking of AH peptides is a potentially promising way to stabilize the α-helix structure based on a preorganization effect.^41^ AH peptides are expected to be useful scaffolds for the design of not only fluorogenic probes but also various functional probes by combining suitable signaling units with a view toward exosome analysis.^42^ We envision that AH peptides also serve as versatile agents to capture exosomes when isolating and purifying the complex samples.^43^ We are continuing studies in these directions.

## Conflicts of interest

There are no conflicts to declare.

## Supplementary Material

RA-010-D0RA07763A-s001
