# Supplementary material for: Amphipathic helical peptide-based fluorogenic probes for a marker-free analysis of exosomes based on membrane-curvature sensing
Source: RSC Adv. 2020 Oct 19;10(63):38323–7. doi: 10.1039/d0ra07763a (PMC9057301; doi:10.1039/d0ra07763a)
Supplement: RA-010-D0RA07763A-s001 [file RA-010-D0RA07763A-s001.pdf]

Electronic Supplementary Material (ESI) for RSC Advances.
This journal is © The Royal Society of Chemistry 2020

Supporting information for

Amphipathic helical peptide-based fluorogenic probes for a marker-free exosome analysis based on membrane-curvature sensing

Yusuke Sato,[a,b]* Kazuki Kuwahara,[a] Kenta Mogami,[a] Kenta Takahashi[a] and Seiichi Nishizawa[a]

[a]Department of Chemistry, Graduate School of Science, Tohoku University, 6-3 Aramaki-Aza Aoba, Aoba-ku, Sendai, 980-8578, Japan.

[b]JST, PRESTO, 4-1-8 Honcho, Kawaguchi, Saitama, 332-0012, Japan.

*Corresponding authors: E-mail: yusuke.sato.a7@tohoku.ac.jp Telephone: +81 22 795 6551

Contents

1. Structures of the fluorescent probes (Fig. S1)
2. HPLC profiles for the probe purification (Fig. S2)
3. Probe characterization (Table S1)
4. Fluorescence response of a control NR molecule to synthetic vesicles (Fig. S3)
5. Titration curves of the binding of ApoC-NR to synthetic vesicles (Fig. S4)
6. Fluorescence response of ApoC-NR for DOPC/DOG vesicles and α- helix contents in the bound state (Fig. S5)
7. Fluorescence response of ApoCmut-NR to synthetic vesicles (Fig. S6)
8. Fluorescence response of ApoC-NBD to synthetic vesicles (Fig. S7)
9. Comparison of the dissociation constants of peptide-based probes (Table S2)
10. Size distribution profiles of exosomes used in this study (Fig. S8)
11. CD spectral change of ApoC-NR upon binding to exosome (Fig. S9)
12. Fluorescence response of ApoC-NR to various exosome (Fig. S10)

Experimental

General: Fmoc-protected amino acids were purchased from Watanabe Chemical Industries (Hiroshima, Japan) or AAPPTec (Louisville, KY, U.S.A.). All phospholipids (phoshpholipids 1-palmitoy-2-oleyl-sn-glycero-3-phosphoethanolamine (POPE), 1-palmitoy-2-oleyl-sn-glycero -3-phospho-L-serine (POPS), 1-palmitoy-2-oleyl-sn-glycero-3-phosphocholine (POPC), 1,2- dioleoyl-sn-glycero-3-phosphocholine (DOPC), 1-2-dioleoyl-sn-glycerol (DOG)) and cholesterol were purchased from Avanti Lipids (Alabaster, AL, USA). Nile Red derivatives (6-(9-diethylamino-5-oxo-5*H*-benzo[*a*]phenoxazin-2-yloxy)hexanoic acid and 2-butoxy-9- (diethylamino)-5H-benzo[a]phenoxazin-5-one) and an NBD derivative (6-((7-nitrobenzo[c][1,2,5]oxadiazol-4-yl)amino)hexanoic acid) were synthesized according to the literature.[(S1)-(S4)] Other reagents were commercially available and used without further purification. Exosomes were purchased from HansaBioMed Life Sciences (Tallinn, Estonia). Water was deionized (≥18.0 MΩ cm specific resistance) by an Elix 5 UV water purification system and a Milli-Q Synthesis A10 system (Millipore Corp., Bedford, MA, USA), followed by filtration through a BioPak filter (Millipore Corp.). Unless otherwise mentioned, all measurements were performed at 25°C in 1×PBS buffer solution (pH 7.4). Errors are the standard deviations obtained from three independent experiments (N = 3).

Preparation of lipid vesicles: Synthetic vesicles were prepared by extrusion according to the literature (Ref. 32 in the main text). Chloroform suspended POPC, POPE, POPS and cholesterol were combined to form lipid mixtures of the desired molar ratio. The lipid film was obtained by drying under nitrogen gas and then hydrated overnight at 4°C in PBS buffer (pH 7.4). The lipid suspension was subjected to five freeze-thaw cycles (except for extrusion with 400 and 1000 nm pores). The vesicles were then prepared by manual extrusion through polycarbonate membranes (Whatman, NJ, USA) with pore diameters of 100, 400, 1000 nm using Avanti mini-extruder (Avanti lipids), which affords the average diameter of 110 nm (V_{110}), 350 nm (V_{350}), and 650 nm (V_{650}), respectively. Vesicle size was characterized by dynamic light scattering (DLS) measurements (Zetasizer Nano-ZS, Malvern, UK).

Synthesis of peptide probe: All probes were synthesized using Biotage Initiator+ microwave peptide synthesizer (Biotage, Uppsala, Sweden) based on a Fmoc solid phase peptide chemistry on a Rink-Amide-ChemMatrix resin (Biotage). 1-[(1-(cyano-2-ethoxy-2-oxoethylideneamino-oxy)-dimethylamino-morpholino methylene)]methana minium hexafluorophosphate (COMU) /

diisopropylethylamine (DIEA) system was employed for the coupling reaction. After completion of the elongation of all amino acid residue units, 6-(9-diethylamino-5-oxo-5*H*-benzo [*a*]phenoxazin-2-yloxy)hexanoic acid was introduced at N-terminus by incubation for 2 × 24h at room temperature. The deprotection of the peptides and the cleavage from the resin were conducted using trifluoroacetic acid/triisopropylsilane/water (95/2.5/2.5). The solution was dropped into cold diethyl ether in order to precipitate the crude peptide probe. The obtained crude product was purified by a reverse-phase HPLC system (pump, PU-2086 Plus ×2; mixer, MX 2080-32; column oven, CO-1565; detector, UV-2070 plus and UV-1570M (Japan Spectroscopic Co. Ltd., Tokyo, Japan)) equipped with a C18 column (Inertsil ODS3; GL Sciences Inc., Tokyo, Japan) using a gradient of water/acetonitrile containing 0.3% TFA (Fig. S3). The probe was verified by MALDI-TOF-MS (Bruker Daltonics autoflex Speed-S1, Germany).

The concentration of ApoC-NR and ApoCmut-NR was determined based on the molar absorption coefficient of 6-(9-(diethylamino-5-oxo-5*H*-benzo[a]phenoxazin-2-yloxy)-hexanoic acid at 550 nm in MeOH (ε = 30423 $cm^{-1}M^{-1}$).[S5] The concentration of ApoC-NBD was determined based on the molar absorption of N-butyl-7-nitrobenzo[c][1,2,5]oxadiazol-4-amine at 464 nm in DMSO (ε = 20574 $cm^{-1}M^{-1}$).

Fluorescence measurements: Fluorescence spectra and anisotropies were recorded on a JASCO model FP-6500 spectrofluorophotometer (Japan Spectroscopic Co. Ltd., Tokyo, Japan) with a thermoelectrically temperature-controlled cell holder. Measurements were done using a 3 × 3 mm quartz cuvette. As for the spectra measurements, polarizers (excitation and emission polarization set to horizontal and vertical, respectively) were used in order to avoid the effect of light scattering by the vesicles. [S6]

CD measurements: CD spectra were measured with a JASCO model J-800 spectropolarimeter equipped with a thermoelectrically temperature-controlled cell holder (Japan Spectroscopic Co. Ltd., Tokyo, Japan) using a 2 × 10 mm quartz cuvette (optical path length: 10 mm).

Exosome analysis: Commercially available exosomes derived from various cell lines were utilized, in which they were purified by a combination of tangential flow filtration and size exclusion chromatography according to the manufacturer's product information. The concentration and particle size of exosomes were determined using a NanoSight NS300 (Malvern). Then, the exosomes with exact concentrations were used for the examination with ApoC-NR.

ApoC-NR : NR-PVLES FKVSF LSALE EYTKK LN

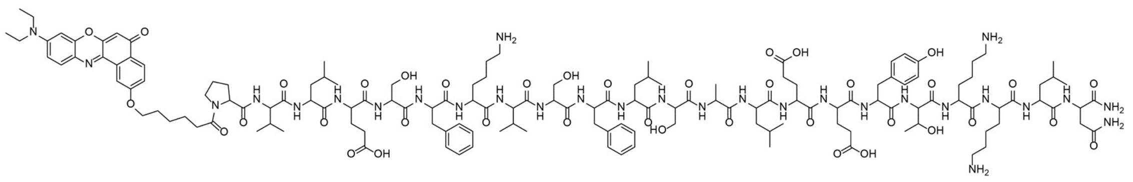

ApoCmut-NR : NR-PVLES FKVSF PSALE EYTKK LN

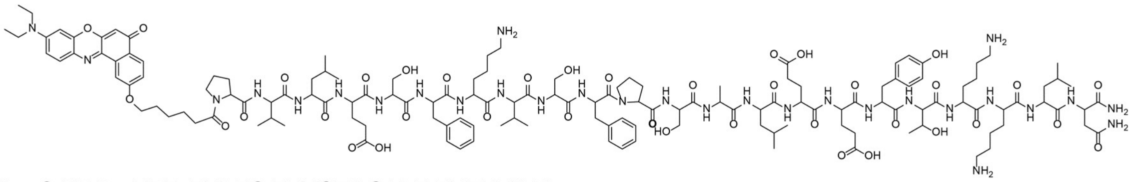

ApoC-NBD : NBD-PVLES FKVSF LSALE EYTKK LN

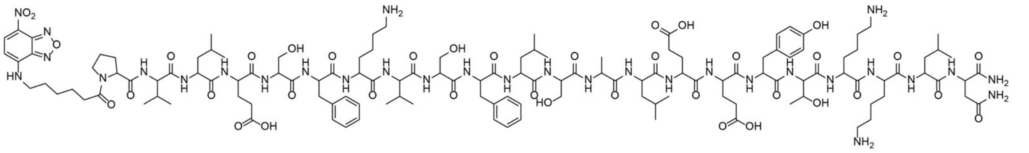

Figure S1. Sequences and chemical structures of peptide probes used in this study.

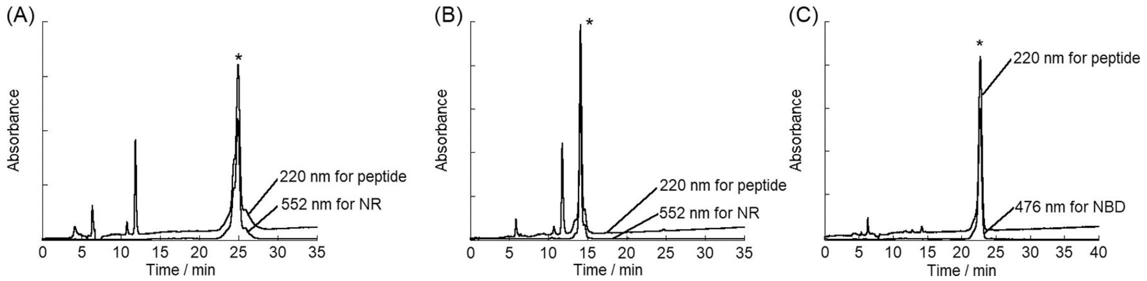

Figure S2. HPLC profile for the purification of (A) ApoC-NR, (B)ApoCmut-NR and (C) ApoC-NBD. Gradient condition: (A),(B) 50-65% CH_3CN (0.3% TFA) in H_2O (0.3% TFA) during 35 min, (C) 50-58% CH_3CN (0.3% TFA) in H_2O (0.3% TFA) during 40 min. Absorbance at 220 nm, 476 nm and 552 nm for the peptide, NBD and NR unit was monitored. The peak (*) was for collected and identified as the purified probe by MALD-TOF-MS.

Table S1. Probe characterization

	Observed mass (m/z)	Calculated mass $[M+H]^+$	Retention time

ApoC-NR	2971.47	2971.58	25.0 min
ApoCmut-NR	2955.68	2955.55	14.0 min
ApoC-NBD	2817.72	2817.48	22.7 min

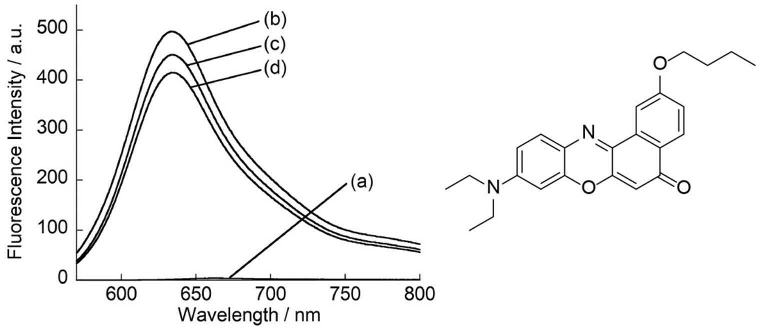

Figure S3. Fluorescence spectra of a free NR molecule without peptide units in the (a) absence and presence of (b) V_{110}, (c) V_{350}, and (d) V_{650}. [NR molecule] = 2.0 μM, [Total lipid] = 500 μM. Temperature, 25°C. Excitation, 552 nm.

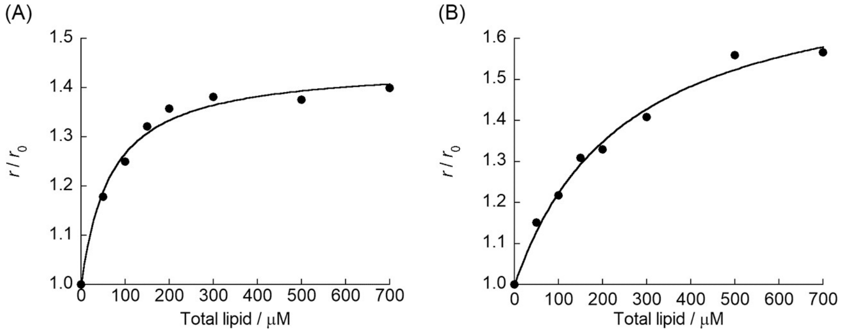

Figure S4. Titration curves for the binding of ApoC-NR to (A) V_{350} and (B) V_{650}. [ApoC-NR] = 2.0 μM. r and r_0 denote the fluorescence anisotropy of ApoC-NR in the presence of synthetic vesicles and absence of the vesicles, respectively. The obtained curves were analyzed with the fitting equation (ref. 22 in the main text).

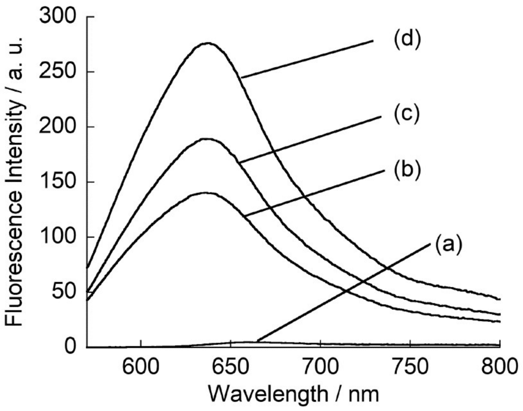

Vesicle	α-helix content
DOPC: 100 %	26%
DOPC: 90%, DOG: 10%	36%
DOPC: 80%, DOG: 20%	51%

Figure S5. Fluorescence spectra of ApoC-NR in the (a) absence and presence of DOPC/DOG vesicles ((b) DOPC: 100% (Diameter 106 nm) ; (c) DOPC: 90%, DOG: 10% (Diameter 106 nm); (d) DOPC: 80%, DOG: 20% (Diameter 107 nm)). α-Helix content of ApoC-NR in the bound state with DOPC/DOG vesicles was also shown, as determined from CD experiments (cf. Fig. S4).

[ApoC-NR] = 2.0 μM, [Total lipid] = 100 μM.

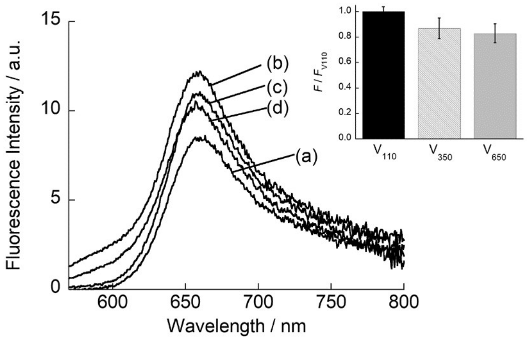

Figure S6. Fluorescence spectra of ApoCmut-NR in the (a) absence and presence of (b) V_{110}, (c) V_{350}, and (d) V_{650}. Inset: Selectivity of ApoCmut-NR for the vesicle size. F and F_{V110} denote the fluorescence intensity of the probe in the presence of synthetic vesicles and V_{110}, respectively. Excitation, 552 nm. Analysis, 660 nm. $N = 2$

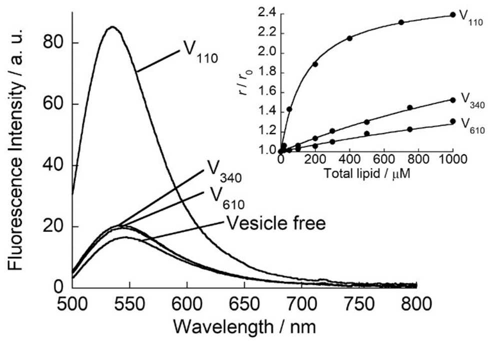

Figure S7. Fluorescence response of ApoC-NBD (2.0 μM) for synthetic vesicles (1.0 mM) with 110 nm (V_{110}), 340 nm (V_{340}) and 610 nm (V_{610}) in size (Lipid composition: 50% POPC, 15% cholestereol, 15% POPE, 20% POPS). Inset: Titration curves for the binding of ApoC-NBD to synthetic vesicles. Excitation, 480 nm. Analysis, 545 nm.

K_d value for V_{100} was estimated as 186 ± 38 μM whereas the affinity for V_{340} and V_{610} was not accurately determined due to the weak binding (K_d > 1500 μM). The observed selectivity of ApoC-NBD to membrane curvature of the vesicles is almost comparable to that of ApoC-NR (Fig. 3 and Table S1).We noticed that the affinity of ApoC-NBD was 31-fold weaker compared to ApoC-NR. This may result from the looping up of NBD moiety.[S7] Meanwhile, NR moiety tends to be embedded in the membrane due to its hydrophobicity,[S7] it would contribute to the stronger binding of ApoC-NR.

Table S2. The apparent dissociation constants (K_d) for synthetic vesicles of ApoC-NR and peptide probes reported in the literatures (MARCKS-ED-NBD,C2BL3C probe and bradykinin probe).

(A) 20% POPS-containing vesicles

ApoC-NR[a]		MARCKS-ED-NBD[a,1]	
Vesicle Size	K_d / μM	Vesicle Size	K_d / μM
110 nm	4.7 ± 0.61	43 nm	16 ± 3
350 nm	82 ± 12	124 nm	18 ± 1
650 nm	100 ± 8.2	439 nm	26 ± 6

C2BL3C probe[b,2]		Bradykinin probe[a,3]	
Vesicle Size	K_d / μM	Vesicle Size	K_d / μM
30 nm	520 ± 200	58 nm	407 ± 56
100 nm	> 1000	116 nm	893 ± 101
400 nm	> 1000	454 nm	902 ± 141

(B) 0% POPS-containing vesicles

ApoC-NR[c]		MARCKS-ED-NBD[c,1]	
Vesicle Size	K_d / μM	Vesicle Size	K_d / μM
110 nm	5.8 ± 1.0	46 nm	141 ± 17
390 nm	46 ± 17	121 nm	64 ± 5
920 nm	> 300	438 nm	146 ± 18

C2BL3C probe[d,2]		Bradykinin probe[c,3]	
Vesicle Size	K_d / μM	Vesicle Size	K_d / μM
30 nm	510 ± 100	58 nm	996 ± 230
100 nm	> 1000	116 nm	940 ± 160
400 nm	> 1000	454 nm	714 ± 70

[1]Data from ref. 30 in the main text. [2]Data from ref. 31 in the main text. [3]Data from ref. 32 in the main text. [a]Lipid composition: 50% POPC, 15% cholestereol, 15% POPE, 20% POPS. [b]Lipid composition: 80% POPC, 20% POPS. [c]Lipid composition: 70% POPC, 15% cholestereol, 15% POPE, 0% POPS. [d]Lipid composition: 100% POPC, 0% POPS.

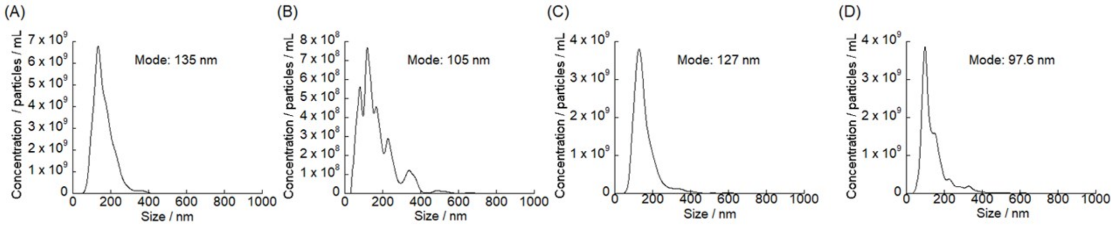

Figure S8. Size distribution profiles of (A) Exo-K562, (B) Exo-BPH-1, (C) Exo-U87MG and (D) Exo-A549, obtained by nanotracking analysis.

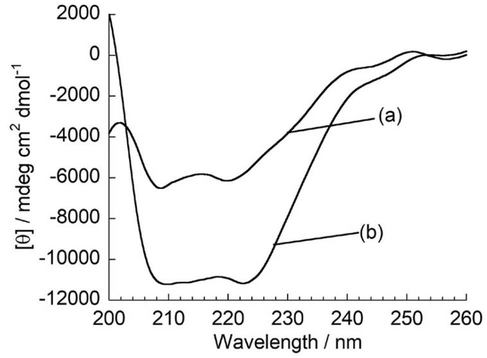

Figure S9. CD spectra of ApoC-NR (2.0 μM) in the absence and presence of Exo-K562 (5.0×10^6 particles/μL).

It was found that α-helix content of ApoC-NR increased from 17% to 33% upon binding to Exo-K562.

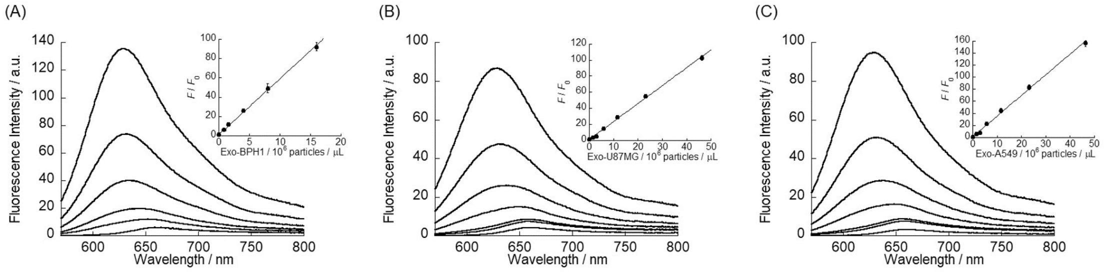

Figure S10. Fluorescence response of ApoC-NR (2.0 μM) for (A) Exo-BPH-1, (B) Exo-U87MG and (C) Exo-A549. Inset: Calibration curve of Exo-K562 based on the fluorescence response (F/F_0) of ApoC-NR. F and F_0 denote the fluorescence intensity of ApoC-NR at 620 nm in the presence and absence of exosomes, respectively. Excitation, 552 nm.

References

(S1) Leitao, M. I. P. S.; Raju, B. R.; Naik, S.; Coutinho, P. J. G.; Sousa, M. J.; Goncalves, M. S. T. *Tetrahedron Lett.* **2016**, 3936-3941.

(S2) Pinkert, T.; Furkert, D.; Korte, T.; Herrmann, A.; Arenz, C. *Angew. Chem. Int. Ed.* **2017**, 56, 2790-2794.

(S3) Hong, Y-R.; Lam, C. H.; Tan, K-T. *Bioconjugate Chem.* **2017**, 28, 2895-2902.

(S4) Storz, M. P.; Allegretta, G.; Kirsch, B.; Empting, M.; Hartmann, R. W. *Org. Biomol. Chem.*, **2014**, 12, 6094-6104.

(S5) Briggs, M. S. J.; Bruce, I.; Miller J. N.; Moody, C. J.; Simmonds, A. C.; Swann, E. *J. Chem. Soc. Perkin Trans.* **1997**, 1, 1051-1058.

(S6) Ladokhin, A. S.; Jayasinghe, S.; White, S. H. *Anal. Biochem.* **2000**, 285, 235-245.

(S7) Saxena, R.; Shrivastava, S.; Haldar, S.; Klymchenko, A. S.; Chattopadhyay, A. *Chem. Phys. Lipids* **2014**, 183, 1-8.
